# 

**Published:** 1864-05

**Authors:** 


Vol. V. MAY, 1864. No. 5.

### THE CHICAGO MEDICAL EXAMINER

A MONTHLY JOURNAL, DEVOTED TO THE

EDUCATIONAL, SCIENTIFIC, AND PRACTICAL INTERESTS

OF THE

MEDICAL PROFESSION.

EDITED BY

N. S. DAVIS, M.D.,

PROFESSOR OF PRINCIPLES AND PRACTICE OF MEDICINE AND OF CLINICAL MEDICINE, IN THE MEDICAL DEPARTMENT OF LIND UNIVERSITY, ETC., ETC.

TERMS, $2.00 PER ANNUM, IN ADVANCE.

CHICAGO:
GEORGE H. FERGUS, BOOK AND JOB PRINTER,
179 STATE STREET.

